# Research on lncRNA CTBP1-DT as a potential therapeutic target to regulate cell function in colorectal cancer

**DOI:** 10.1007/s12672-024-01085-y

**Published:** 2024-06-12

**Authors:** Ruizhi Fan, Teng Xu, Yuting Kuang

**Affiliations:** 1 Department of Gastrointestinal Surgery, The First Affiliated Hospital of Soochow University, No.188, Shizi Street, Suzhou, 215006 Jiangsu China; 2 Department of Gastrointestinal Surgery, The Affiliated Hospital of Xuzhou Medical University, Xuzhou, 221004 Jiangsu China

**Keywords:** Colorectal cancer, lncRNA CTBP1-DT, miR-30a-5p, Diagnosis, Silencing CTBP1-DT

## Abstract

**Background:**

Colorectal cancer, which originates from the human colon or rectum, is one of the leading causes of death worldwide. Timely diagnosis and interventional therapy can significantly improve the prognostic survival of colorectal cancer patients, making regular screening and early detection essential.

**Aim:**

To investigate the regulatory function of lncRNA CTBP1-DT (CTBP1-DT) on colorectal cancer cells and to assess its diagnostic significance.

**Methods:**

A total of 102 patients with colorectal cancer and 92 healthy individuals were selected. The levels of CTBP1-DT and microRNA-30a-5p (miR-30a-5p) in serum and cell samples of the above subjects were compared by RT-qPCR. The effects of CTBP1-DT and miR-30a-5p dysregulation on the biological functions of colorectal cancer cells were analyzed via CCK-8, flow cytometry and Transwell assays. In addition, the ability of CTBP1-DT and miR-30a-5p to early identify colorectal cancer patients was determined through ROC curve.

**Results:**

Serum CTBP1-DT was elevated in patients with colorectal cancer, which was obviously higher than in healthy controls. The expression of serum miR-30a-5p was downregulated in colorectal cancer. Both CTBP1-DT and miR-30a-5p have the value of distinguishing colorectal cancer, and the combined diagnostic ability is higher. Knockdown of CTBP1-DT directly targeted miR-30a-5p to repress cell activity and metastatic ability, whereas deregulation of miR-30a-5p eliminated the above inhibitory effects.

**Conclusion:**

Overexpression of CTBP1-DT has a certain application potential in the diagnosis of colorectal cancer and may be a therapeutic target for colorectal cancer.

## Introduction

Colorectal cancer is a cancer of the digestive tract originating from the epithelial mucosa of the large intestine that threatens human life and health [1]. Early stages of colorectal cancer have insidious development and lack of specificity in clinical manifestations, resulting in a low rate of early diagnosis of colorectal cancer, and most of the patients have already reached the middle or late stage when diagnosed [2]. Despite the continuous improvement of medical technology, the survival outcomes of patients with early diagnosis of colorectal cancer have been greatly improved, but the survival rate of patients with advanced colorectal cancer is still very low, making colorectal cancer a heavy burden of social medical care [3, 4]. Therefore, finding effective diagnostic indicators for early colorectal cancer and discovering the relationship between abnormal levels and tumor cell function are of great significance for the intervention and targeted therapy of patients.

Long non-coding RNA (lncRNA) is a class of nucleotide transcripts without protein coding function, which are involved in numerous physiological processes such as epigenetic inheritance, cellular activity, and gene regulation [5–7]. In the advancement of colorectal cancer research, lncRNA ITGB8-AS1 was considered as a useful target to curb tumor progression by mediating focal adhesion signaling [8]. In addition, lncRNA SNHG15 and MSTO2P were elucidated as possible targets for prevention and therapeutic targets in colorectal cancer [9, 10]. CTBP1-DT is located on human chromosome 4p16.3, which was emphasized to be associated with tumorigenesis [11]. For example, CTBP1-DT may be a prognostic risk factor for head and neck squamous cell carcinoma as a ferroptosis-related lncRNA [12]. The potential of CTBP1-DT in cisplatin resistance in ovarian cancer was suggested by Ren et al. [11]. However, CTBP1-DT has been less studied in tumor therapy and detailed evidence on the role of CTBP1-DT in colorectal cancer is still lacking. Meanwhile, microRNAs (miRNAs) are also of exploratory interest in tumors. Numerous studies revealed the involvement of dysregulated miR-30a-5p in the progression of colorectal cancer [13–15].

In this study, abnormally expressed lncRNAs in colorectal cancer was predicted using the GEO online database and CTBP1-DT was found to be overexpressed in colorectal cancer. Furthermore, the possible downstream targets of CTBP1-DT were evaluated by bioinformatics website. The diagnostic potential of CTBP1-DT combined with miR-30a-5p was explored by verifying the expression of CTBP1-DT and miR-30a-5p in colorectal cancer, with the aim of bringing a new direction for the discovery of potential markers of colorectal cancer.

## Methods

### Enrollment of patients

Colorectal cancer patients (n = 102) attending The First Affiliated Hospital of Soochow University from January 2022 to October 2023 were selected for the study subjects and healthy individuals (n = 92) were included as controls. Inclusion criteria: (1) All patients were confirmed by pathology experts for the first time and received corresponding treatment; (2) Patients did not receive radiotherapy or chemotherapy before the experiment; (3) All patients were adult without pregnancy; (4) Informed consent was obtained from patients and guardians; (5) Patients had no previous history of colorectal cancer or other major diseases. Exclusion criteria: (1) People with other tumors or serious diseases; (2) People with systemic diseases or family genetic diseases; (3) People who are unable to take care of themselves or unwilling to participate in the study; (4) Patients with a history of antitumor therapy for colorectal cancer; (5) Patients without complete clinical information. The Ethics committee of The First Affiliated Hospital of Soochow University reviewed and approved this study.

### Serum sample collection

Venous blood specimens were collected in early morning fasting state in anticoagulant-free centrifuge tubes from both included colorectal cancer patients and healthy individuals. The venous blood was allowed to stand for 10 min and then centrifuged at room temperature, with the centrifuge set at 3000 r/min for 15 min. The serum obtained was stored in centrifuge tubes and frozen in a -80°C refrigerator.

### Online analysis of bioinformatics

The abnormal expression of lncRNAs in colorectal cancer was retrieved from the GEO database (GSE249054 dataset). The potential downstream miRNAs of CTBP1-DT were predicted according to ENCORI (2023) and DIANA (2019) online websites, and Venn diagram was made. Meanwhile, the linked sites between miR-30a-5p and CTBP1-DT were queried through the ENCORI website.

### Gene expression assays

The RNA solution was obtained by transferring Trizol reagent (Sigma-Aldrich, USA) in serum and cell samples and mixing by repeated blowing, then adding chloroform (Shanghai Acmec Biochemical, China) and isopropanol (Aladdin, China) sequentially. The concentration of RNA was measured using a spectrophotometer, and the RNA was then reverse transcribed into cDNA as required by the PrimeScript RT Mix kit (Takara, Japan). The cDNA was used as a template for real-time quantitative PCR (RT-qPCR) amplification using SYBR Green PCR Kit (Takara, Japan). CTBP1-DT used GAPDH as an internal reference and miR-30a-5p used U6 as an internal reference. Amplification experiments and fluorescence signal detection were performed by an ABI 7500 PCR instrument (Applied Biosystem, USA). The expression of CTBP1-DT and miR-30a-5p was calculated using the 2^−ΔΔCt^ method. The primer sequences were: CTBP1-DT (forward) 5'-TAAGATCGGGGCTGCCGAG-3', (reverse) 5'-TCCCTCCTTCATGACTCCC-3'; miR-30a-5p (forward) 5'-CGATGTTGAAACATCCTCGAC-3', (reverse) 5'-CCAGTGCAGGGTCCGAGG-3'; GAPDH (forward) 5'-TATCGCTGCGCTGGTCGTC-3', (reverse) 5'-AGGATGGCGTGAGGGAGAGC-3'; U6 (forward) 5'-CTCGCTTCGGCAGCACA-3', (reverse) 5'-AACGCTTCACGAATTTGCGT-3'.

### Cell culture and transfection

Human normal colon epithelial cells (NCM460) and colorectal cancer cell lines (HCT15, SW1116, SW837, SNU503) were purchased from the Chinese Academy of Sciences (Shanghai, China). The resuscitated cells were seeded in DMEM medium containing 1% penicillin–streptomycin double antibody (Thermo Fisher Scientific, USA) and 10% fetal bovine serum (FBS; Takara, Japan) and placed in a cell incubator (37°C, 5% CO_2_) for routine culture.

### Luciferase activity assay

The binding sites of CTBP1-DT and miR-30a-5p were amplified to construct wild-type CTBP1-DT (wt-CTBP1-DT) and mutant-type CTBP1-DT (mut-CTBP1-DT). SNU503 cells were co-transfected with wt/mut-CTBP1-DT and mimic/inhibitor NC or miR-30a-5p mimic/inhibitor, and luciferase activity was assessed by the Dual Luciferase Assay Kit (Promega, USA).

### Transfection assay

CTBP1-DT small interfering RNA sequence (si-CTBP1-DT) and negative control sequence (si-NC) were transfected into SNU503 cells by Lipofectamine 2000 transfection reagent when the confluence rate of cell growth reached about 80%. In addition, si-CTBP1-DT and inhibitor NC/miR-30a-5p inhibitor were also co-transfected into SNU503 cells by Lipofectamine 2000 reagent.

### CCK-8 assay

SNU503 cells were inoculated in 96-well plates at a cell density of 3 × 10^3^ cells/well, and 3 replicate wells were set up in each group and routinely cultured. The cell proliferation activity was evaluated by adding CCK-8 reagent (Dojindo, Japan) to each well at 0, 24h, 48h and 72h of cell culture, and the optical density (OD) value at 450 nm was detected by microplate reader (Agilent, USA) after continuing the incubation for 2 h.

### Annexin V assay

Transfected cells were stained according to the requirements of Annexin V-FITC PI Apoptosis Detection kit (BD Biosciences, USA), and the apoptosis rate was characterized by flow cytometry (BD Biosciences, USA).

### Transwell assay

SNU503 cells, which were successfully transfected and growing in logarithmic phase, were resuspended in DMEM medium and the density was adjusted to 3 × 10^5^ cells/well. The above cell suspension was transferred to the top of the Transwell and DMEM medium with 10% FBS was added to the bottom. After routine culture for 48 h, the cells were fixed with methanol and stained with crystal violet, and the number of migrated cells was observed in 5 randomly selected fields of the microscope.

Matrigel (Solarbio, China) was applied above the Transwell chamber, and the level of cell invasion was determined in the same manner.

### Statistical analysis

Data were analyzed by GraphPad 9.0 and SPSS 20.0 software. Count data was expressed as n, and the relationship between abnormal CTBP1-DT level and clinical indicators of patients was tested by chi-square test. Comparisons between two groups were made by *t*-test and comparisons between multiple groups were analyzed by ANOVA. The value of CTBP1-DT and miR-30a-5p for the diagnosis of colorectal cancer was evaluated using ROC curves. To reduce the error of the results, each set of samples was set in three parallel and repeated at least three times. *P* < 0.05 means that the difference is statistically significant.

## Results

### CTBP1-DT in colorectal *cancer* serum

**Fig. 1:**
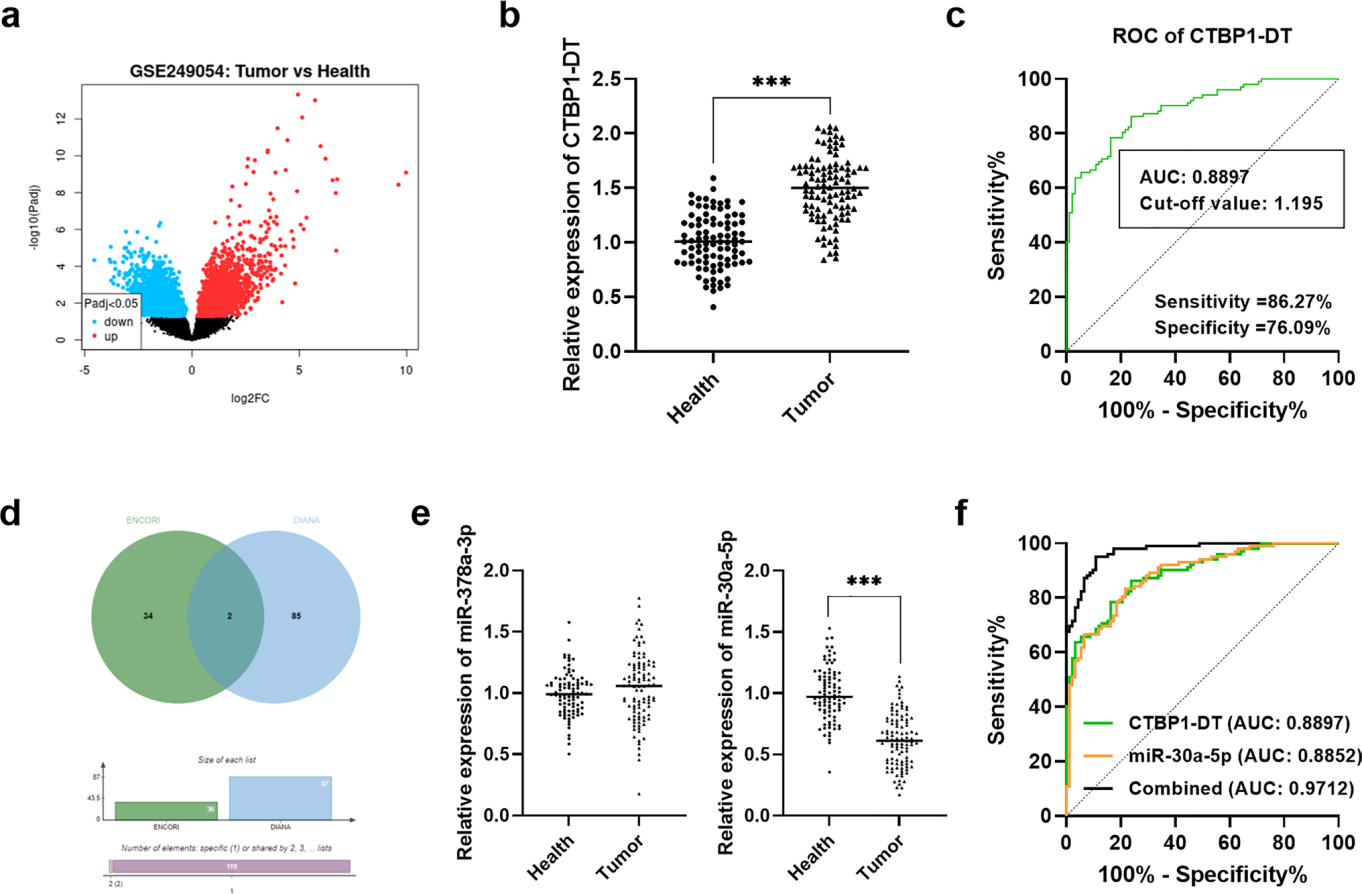
CTBP1-DT and miR-30a-5p expression in colorectal cancer serum. **a** Prediction of abnormally expressed lncRNAs in colorectal cancer by GSE249054 dataset. **b** CTBP1-DT was positively expressed in prostate cancer. **c** Diagnostic value of abnormal levels of CTBP1-DT in colorectal cancer (AUC: 0.8897, cut-off value: 1.195). **d** Venn diagram of the online database for CTBP1-DT downstream target predictions. **e** Detection of miR-378a-3p and miR-30a-5p expression in serum samples. **f** The potential of CTBP1-DT and miR-30a-5p combined to identify colorectal cancer (AUC: 0.9712). (***P < 0.001 vs health)

The upregulated and downregulated lncRNAs in colorectal cancer were analyzed using the GSE249054 dataset (Fig. 1a). The selected CTBP1-DT level in colorectal cancer serum samples were detected by RT-qPCR and found it to be prominently expressed (Fig. 1b). Based on the mean value of CTBP1-DT expression, the patients included were categorized into low-group (n = 50) and high-group (n = 52). When combined with specific clinical indicators, elevated CTBP1-DT was associated with high-grade TNM stage (*P* = 0.030), lymph node metastasis (*P* = 0.016) and positive Ki67 expression (*P* = 0.048), as illustrated in Table 1. Furthermore, ROC curve elaborated that the sensitivity and specificity (86.27% and 76.09%) of CTBP1-DT in identifying colorectal cancer patients were higher than 70%, indicating the high diagnostic potential of CTBP1-DT (AUC: 0.8897, cut-off value: 1.195; Fig. 1c).

**Table 1:** Correlation of the lncRNA CTBP1-DT expression with clinical characteristics in patients with colorectal cancer

Parameters	Cases (n = 102)	Low expression (n = 50)	High expression (n = 52)	*P*
Age				0.432
≤ 60	49	26	23	
> 60	53	24	29	
Gender				0.538
Male	56	29	27	
Female	46	21	25	
Tumor size				0.072
≤ 5 cm	54	31	23	
> 5 cm	48	19	29	
Focal location				0.550
Colon	50	23	27	
Rectum	52	27	25	
TNM stage				0.030
I-II	46	28	18	
III-IV	56	22	34	
Differentiation				0.237
Low	53	23	30	
Medium–High	49	27	22	
Lymph node metastasis				0.016
Negative	57	34	23	
Positive	45	16	29	
Ki67 expression				0.048
Negative	41	25	16	
Positive	61	25	36	

### miR-30a-5p in colorectal *cancer* serum

The Venn diagram reflects that there are two downstream miRNAs are predicted in the results by the online network of ENCORI and DIANA (Fig. 1d). The validation of miR-378a-3p and miR-30a-5p levels in the samples revealed that miR-30a-5p was dysregulated in the serum of colorectal cancer compared with the healthy control group (Fig. 1e). Moreover, the AUC for serum CTBP1-DT and miR-30a-5p in colorectal cancer was 0.8897 and 0.8852, respectively, and the AUC for combined diagnosis was 0.9712 (Fig. 1f).

### Targeting relationship between CTBP1-DT and miR-30a-5p

**Fig. 2:**
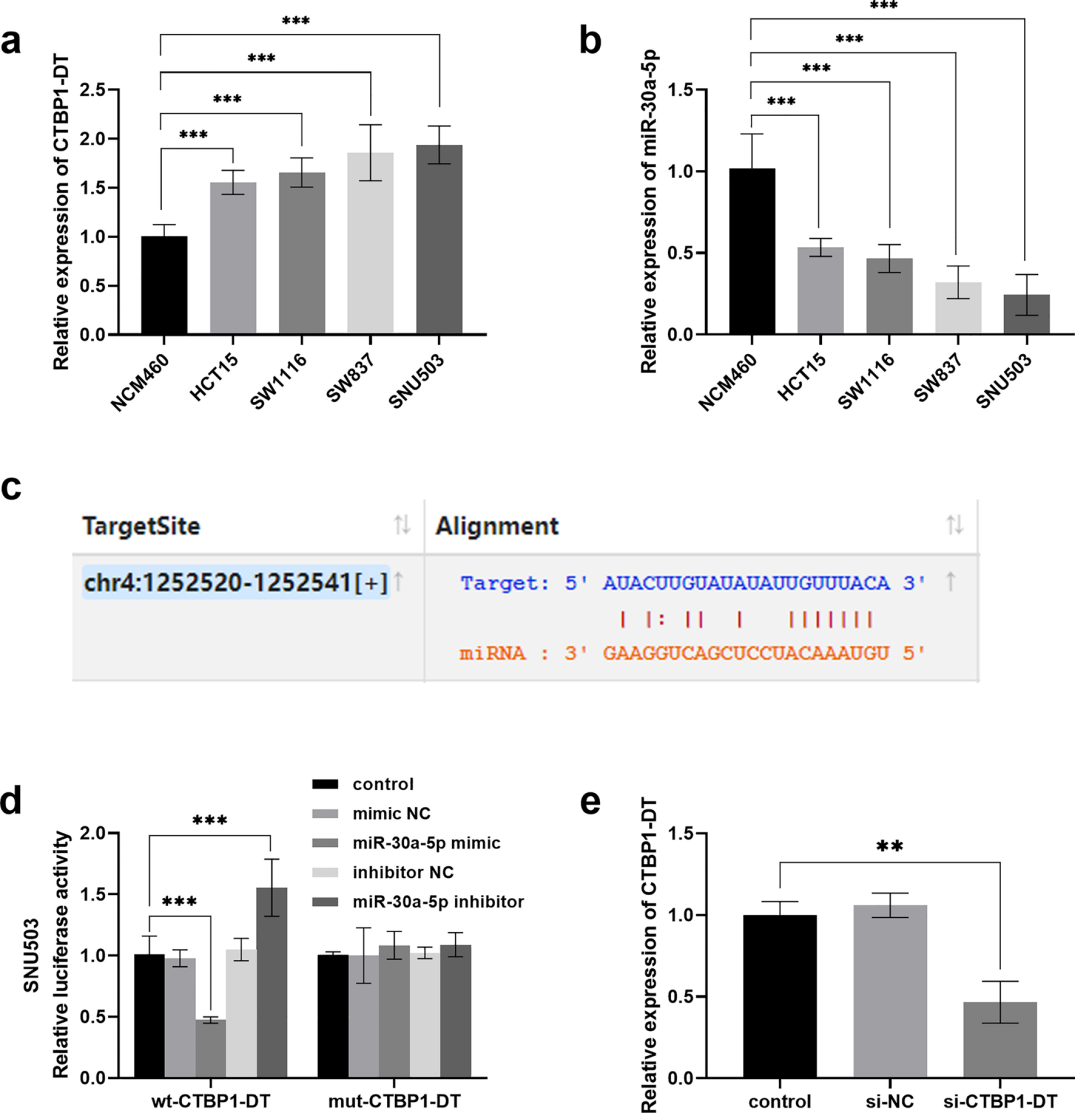
Validation of the CTBP1-DT sponge miR-30a-5p. **a** CTBP1-DT and **b** miR-30a-5p levels in colorectal cancer cells. **c** Linking sites between CTBP1-DT and miR-30a-5p. **d** Assay of luciferase activity in SNU503 cells. **e** After silencing CTBP1-DT by transfection, the amount of CTBP1-DT in the cells was suppressed. (**P < 0.01, ***P < 0.001 vs control)

CTBP1-DT and miR-30a-5p expression was examined at the cellular level, revealing that CTBP1-DT was upregulated while miR-30a-5p was downregulated in colorectal cancer cells (Fig. 2a, b). The ENCORI website revealed binding sites for both CTBP1-DT and miR-30a-5p (Fig. 2c). The luciferase experiment further verified the regulatory impact of CTBP1-DT on miR-30a-5p. In Fig. 2d, the luciferase activity of cells reduced after co-transfection with wt-CTBP1-DT and miR-30a-5p mimic, while increased after transfection with wt-CTBP1-DT and miR-30a-5p inhibitor. Moreover, the CTBP1-DT level in cells significantly decreased after transfection with silencing CTBP1-DT (Fig. 2e).

### Regulation of CTBP1-DT and miR-30a-5p on biological functions of colorectal *cancer* cells

**Fig. 3:**
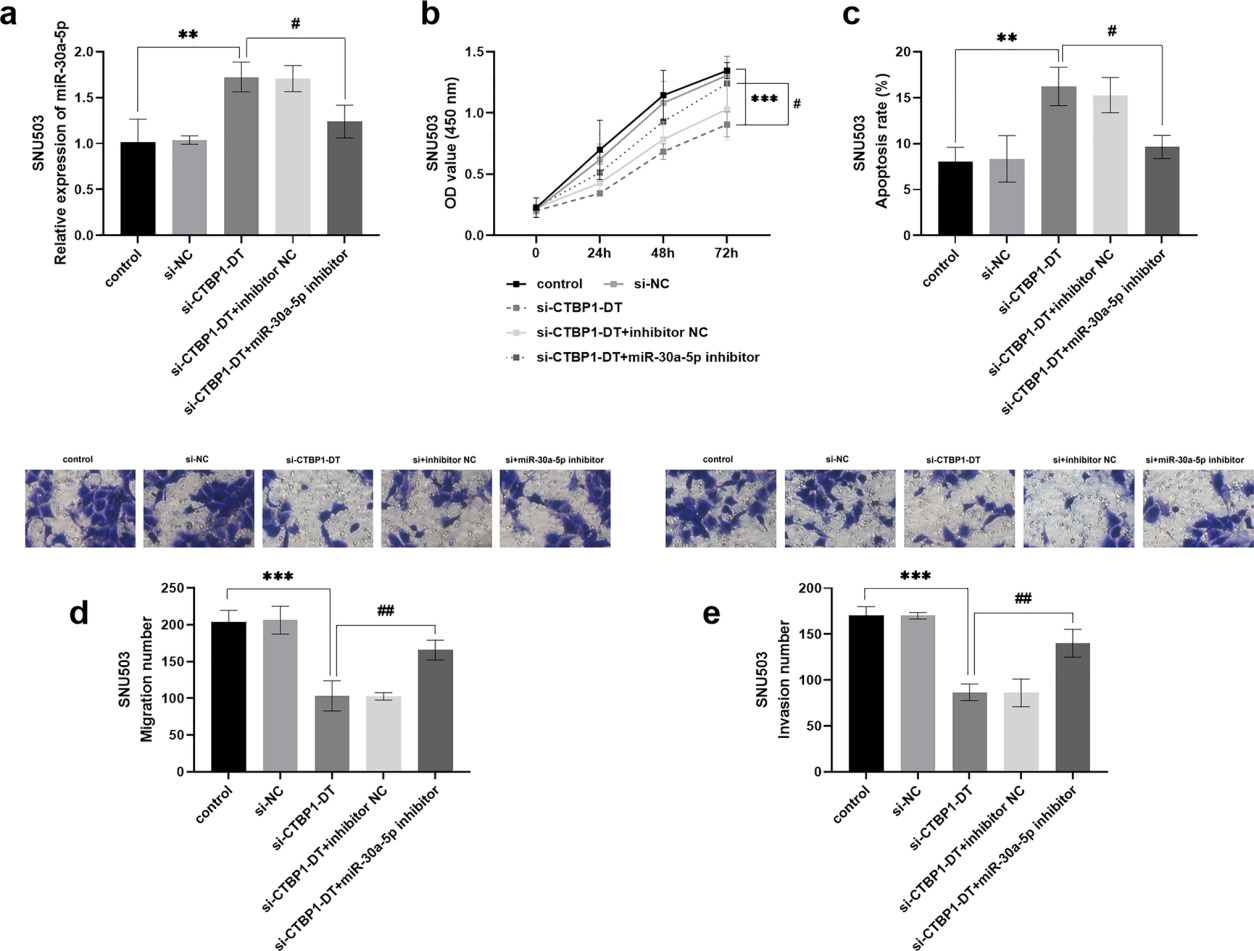
Effect of silencing CTBP1-DT and miR-30a-5p on the viability of colorectal cancer cells. **a** After transfection with silencing CTBP1-DT, the level of miR-30a-5p was up-regulated in cells, while transfection with low expression miR-30a-5p decreased the expression of miR-30a-5p. **b**, **c** si-CTBP1-DT inhibited cell proliferation level and promoted cell apoptosis rate, while miR-30a-5p inhibitor counteracted the negative effect of si-CTBP1-DT on cell growth. **d**, **e** Downregulation of CTBP1-DT reduced the number of cell migration, which was restored by poor expression of miR-30a-5p, as was the level of cell invasion. (**P < 0.01, ***P < 0.001 vs control; ^#^*P* < 0.05, ^##^*P* < 0.01 vs si-CTBP1-DT)

Knockdown of CTBP1-DT enhanced the level of miR-30a-5p in SNU503 cells, but using miR-30a-5p inhibitor restored the miR-30a-5p level (Fig. 3a). Reduced expression of CTBP1-DT slowed the growth of SNU503 cells, yet co-transfection with si-CTBP1-DT and miR-30a-5p inhibitor boosted cell activity (Fig. 3b). Conversely, the cell apoptosis rate was increased after transfection with si-CTBP1-DT, which was reversed by miR-30a-5p inhibitor (Fig. 3c). Figure 3 illustrates that the biological function of SNU503 cells was suppressed when CTBP1-DT was knockdown, but this inhibitory ability was lessened when miR-30a-5p was silenced (Fig. 3d, e).

## Discussion

Colorectal cancer is a prevalent malignant digestive tract tumor in China with a complex and not fully understood pathogenesis [16]. Early treatment can enhance patient outcomes and extend the survival time, potentially reaching a 5-year prognosis survival rate of 90% [17]. As the economy advances, the dietary structure of the population has changed dramatically, leading to an extremely high incidence of colorectal cancer [18, 19]. Evidence suggests that cell proliferation and spread accelerate tumor growth, and the activity of tumor cells may cause functional destruction of adjacent organs [20]. Therefore, studying the pathological mechanism of colorectal cancer and the biological function of cancer cells, it is of guiding significance for the diagnosis and reasonable treatment of patients.

Colonoscopy is an effective tool for colorectal cancer screening, but the complexity and invasiveness make large-scale screening challenging [21]. As a new type of tumor marker, ncRNAs (lncRNAs and miRNAs) have simple detection methods and broad applications, making them a current research hotspot. In exploring tumor therapies, Elina et al. demonstrated that polyphenol natural compounds and maggot larvae are novel targets for immunotherapy of gastric cancer by introducing lncRNAs and combining with systematic biological analyses [22]. A recent study also suggested that lncRNA may serve as a drug target for the complementary treatment of colorectal cancer based on sparassis latifolia and exercise [23]. Additionally, understanding the death pathways of tumors at the cellular level can help develop promising therapeutic approaches [24]. Meanwhile, the application of nanomaterials to explore cancer therapeutic drugs has also been a research hotspot in recent years [25–27].

CTBP1-DT is a novel non-coding RNA and stated as a target for DNA damage-based anticancer therapy [28]. Liu et al. suggested that CTBP1-DT was actively expressed in ovarian cancer, promoting malignant cell biological behavior and accelerating tumor progression [29]. Recent studies clarified that upregulation of CTBP1-DT mediates cellular activity and lipid synthesis in renal clear cell carcinoma, while silencing CTBP1-DT induces apoptosis [30]. In this study, CTBP1-DT was verified to be enriched in colorectal cancer serum and cells, effectively distinguishing patients from healthy individuals. Further correlation analysis confirmed that CTBP1-DT was positively correlated with TNM stage, lymph node metastasis and Ki67 expression in CRC patients. These findings suggest that assessing CTBP1-DT expression may be beneficial for clinical diagnosis and disease analysis of colorectal cancer patients.

miRNAs are known to regulate gene expression, influence the cell cycle, and modulate the tumor microenvironment in cancer [31]. miR-30a-5p was revealed to be poorly expressed in ovarian cancer, lung squamous cell carcinoma and prostate cancer [32–34]. Interestingly, miR-30a-5p was also found to be downregulated in colorectal cancer, indicating its potential as a diagnostic and therapeutic factor [35]. This finding was supported by RT-qPCR assay, which demonstrated decreased miR-30a-5p level. The ROC curve results implied its predictive nature in colorectal cancer. Moreover, the combination of CTBP1-DT and miR-30a-5p has been shown to have higher diagnostic value in colorectal cancer.

In the mechanistic study, miR-30a-5p was confirmed as the direct target of CTBP1-DT. In vitro cell experiments illustrated that a decrease in CTBP1-DT expression led to an increase in miR-30a-5p levels in colorectal cancer cells, suggesting that CTBP1-DT targeted and negatively regulated miR-30a-5p. Moreover, silencing CTBP1-DT negatively impacted cell growth, migration, and invasion capacity, while accelerating apoptosis. miR-30a-5p inhibitor counteracted the suppression of biological behavior of colorectal cancer cells by CTBP1-DT dysregulation. Cheng et al. proposed that miR-30a-5p regulates downstream HSPA5 to participate in colorectal cancer formation and cell activity [13]. KCNQ1OT1 mediated the miR-30a-5p/USP22 axis to inhibit the growth of colorectal cancer cells, thereby regulating tumor progression, as reported by Xian et al. [15]. Therefore, miR-30a-5p may regulate downstream factors involved in colorectal cancer generation and development, which needs to be revealed in our subsequent exploration.

Through the existing assays we confirmed that CTBP1-DT mediates miR-30a-5p to affect the process of colorectal cancer, however there are some limitations that cannot be ignored. The number of volunteers involved was limited, and more sample sizes need to be included in the future. More studies need to be accumulated on the mechanism and function of CTBP1-DT/miR-30a-5p axis in colorectal cancer. Experimental design also needs more perfect control group and related image information to enrich the research content. In addition, there are great challenges in translating clinical results into clinical application.

## Conclusions

In conclusion, CTBP1-DT was highly expressed in CRC, while miR-30a-5p showed a downward trend. In terms of regulatory mechanism, dysregulated CTBP1-DT controlled cell proliferation and activity through sponge miR-30a-5p, while miR-30a-5p inhibitor repaired this inhibition. CTBP1-DT provides a referable experimental basis for the clinical diagnosis and treatment of colorectal cancer through regulating the biological functions of tumor cells.

## Data Availability

All data generated or analyzed during this study are included in this article. Further enquiries can be directed to the corresponding author.
